# Peculiarities of pseudogap in Y_0.95_Pr_0.05_Ba_2_Cu_3_O_7−δ_ single crystals under pressure up to 1.7 GPa

**DOI:** 10.1038/s41598-019-55959-1

**Published:** 2019-12-31

**Authors:** A. L. Solovjov, L. V. Omelchenko, E. V. Petrenko, R. V. Vovk, V. V. Khotkevych, A. Chroneos

**Affiliations:** 1 B. Verkin Institute for Low Temperature Physics and Engineering, NAN of Ukraine, 47 Nauky Avenue, Kharkiv, 61103 Ukraine; 2 V. Karazin Kharkiv National University, 4 Svobody Square, Kharkiv, 61077 Ukraine; 3 Diamond Light Source Ltd., Harwell Science and Innovation Campus, Didcot, OX11 0DE United Kingdom; 4 Department of Materials, Imperial College, London, SW7 2AZ UK; 5 Faculty of Engineering, Environment and Computing, Coventry University, Priory Street, Coventry, CV1 5FB United Kingdom

**Keywords:** Electrical and electronic engineering, Superconducting properties and materials

## Abstract

The effect of hydrostatic pressure up to P = 1.7 GPa on the fluctuation conductivity σ′(T) and pseudogap ∆*(T) in Y_0.95_Pr_0.05_Ba_2_Cu_3_O_7−δ_ single crystal with critical temperature Т_с_ = 85.2 K (at P = 0) was investigated. The application of pressure leads to the increase in T_c_ with dT_c_/dP = +1.82 K∙GPa^−1^ while the resistance decreases as dlnρ(100 K)/dP = −(10.5 ± 0.2) %∙GPa^−1^. Regardless of the pressure, in the temperature interval from T_c_ to T_0_ (~88 K at P = 0) the behaviour of σ′(T) is well described by the Aslamazov – Larkin (AL – 3D) fluctuation theory, and above the T_0_ by the Lawrence – Doniach theory (LD). The Maki-Thompson (MT – 2D) fluctuation contribution is not observed. This indicates the presence of structural defects in the sample induced by Pr. Here it is determined for the first time that when the pressure is applied to the Y_1−x_Pr_x_Ba_2_Cu_3_O_7−δ_ single crystal, the pseudogap increases as dlnΔ*/dP = 0.17 GPa^–1^.

## Introduction

The pseudogap (PG) state, which is realized in high-temperature superconductors (HTSCs) at the characteristic temperature T* » T_c_ with the doping less than optimal, is one of the most mysterious properties of HTSC cuprates^1–9^. Understanding the physics of the PG would answer the question about the mechanism of superconducting pairing in HTSCs, which is also not fully clarified yet^3–7,10,11^. One of the most promising materials for studying the PG is the YBa_2_Cu_3_O_7−δ_ (YBCO) family. It is so due to the possibility of variation in its composition by substitution of yttrium (Y) with the isovalent analogues, or changing the degree of oxygen nonstoichiometry (see review^12^ and references therein). The compounds Y_1−х_Pr_x_Ba_2_Cu_3_O_7−δ_ (YPrBCO) with partial substitution of Y by praseodymium (Pr) atoms are of particular interest in that respect. The replacement of Y with other rare-earth elements in this compound does not lead to a significant change in its resistive characteristics^13^. The only exception is the replacement of Y with Pr (also known as the “praseodymium anomaly”), which leads to a noticeable increase in the resistivity ρ and a decrease in the critical temperature T_c_ of the superconducting (SC) transition^14–18^. It is believed that in YPrBCO this occurs as a result of the interaction of holes with electrons of the 4 f shell of Pr. Finally, with increase in Pr content (regardless of the oxygen content), in PrBa_2_Cu_3_O_7−δ_ (PrBCO) the charge carriers are localized in the Ferenbacher-Rice energy zone (RF)^19^. Thus, PrBCO becomes a dielectric being isostructural to YBCO^16–18^. The PrBCO dielectric inclusions, arising in the process of manufacturing of YPrBCO crystals, form multiple defects in the YBCO superconducting matrix^15–18^, which significantly affect the transport properties of the sample. Therefore, doping of the Y_1−х_Pr_x_Ba_2_Cu_3_O_7−δ_ with Pr on the one hand leads to a gradual suppression of superconductivity with increasing of the concentration x, and on the other, it allows to preserve almost constant both the lattice parameters and the oxygen content (7-δ) of the sample under investigation^18–20^. It should also be stressed that Pr^+3^ atoms have an intrinsic magnetic moment of μ_Pr_ ≈ 3.58μ_B_^21^, which is m_eff_ ≈ 2μ_B_ in the PrBCO compound^22^. That is why the study of the effect of Pr impurity on the properties of YPrBaCuO single crystals is considered very promising for revealing the mechanisms of the interplay of the superconductivity and magnetism in HTSC, (for example^23,24^ and references given therein), which is important for the final clarification of the physical nature of both the PG and high-temperature superconductivity in general^1,2,5–7,10,11^.

Hydrostatic pressure is an effective tool for studying HTSCs (refer to^12^ and references therein), which enables validating the adequacy of numerous theoretical models, as well as establishing most significant parameters of HTSC structures, that determine their physical characteristics in normal and SC states.

In cuprates the dT_c_/dP dependence is mostly positive, while the derivative dlnρ/dT is negative and is relatively large^25–31^. However, the data given in studies of the effect of pressure on the T_c_ of YPrBCO compounds (see, for example, Reviews^12,32^) are often inconsistent. The registration of both positive and negative baric derivative dТ_с_/dP is reported, and in some cases the sign change of the dT_c_/dP^12,32^ takes place. Importantly, a significant part of the experimental data was obtained on ceramics, films, and textured samples of very different processing history^13–17,32^. In the case of single-crystal samples, difficulties may be caused by the appearance in the system of a sufficiently disordered structure of twin boundaries (TB)^33,34^. TB being extended two-dimensional defects can serve as drains for lower dimensionality defects, which in turn represent strong scattering centers for normal and fluctuation charge carriers^12,35^, thereby having a significant impact on charge transfer processes in a particular experimental sample.

Notably, in the literature there are no experimental data on the effect of pressure in compounds with Pr concentration x < 0.1. It is the weakly Pr doped samples that demonstrate interesting phenomena of suppression of the pseudogap state and anomalous extension of the temperature range of the linear ρ(T) dependence^18,24,36^. When pressure is applied, the volume of the unit cell decreases contributing to the ordering of the system leading to a decrease in the number of structural defects and a decrease in ρ^12,28,37^. At any rate, the mechanisms of influence of pressure on both T_c_ and ρ are not fully understood, as the nature of the transport properties of HTSC is not completely clear. The main contribution to the conductivity of cuprates is by the CuO_2_ planes, between which there is a relatively weak interplanar interaction. It is assumed that the pressure leads to an increase in the density of charge carriers n_f_ in the conducting CuO_2_ planes and, as a result, to a decrease in ρ^12,28^. An increase in n_f_ under pressure should also lead to an increase in T_c_, i.e. to a positive dT_c_/dP value as observed in experiments^25,27,31,38,39^. Nevertheless, there are only very few studies that investigated the effect of pressure on the fluctuation conductivity and PG in HTSC cuprates^12,38,39^, and, as far as we know, in the YPrBCO such studies have not been carried out at all.

In the present study we investigated the effect of hydrostatic pressure up to P = 1.7 GPa on resistive characteristics, the excess conductivity σ′(T) and the pseudogap ∆*(T) of doped with Pr (x ≈ 0.05) single crystals of Y_1−х_Pr_x_Ba_2_Cu_3_O_7−δ_ with nearly stoichiometric oxygen content. The geometry of the transport current I flow was chosen parallel to the TB (I || TB), which made it possible to minimize the effects of scattering on the twin boundaries^30,40^.

## Results and Discussion

### Resistive characteristics

**Figure 1:**
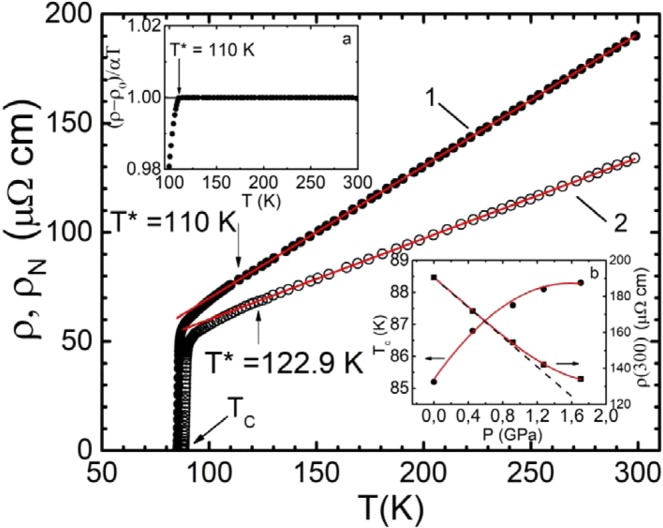
Temperature dependences of the resistivity ρ of Y_0.95_Pr_0.05_Ba_2_Cu_3_O_7–δ_ single crystal at P = 0 (curve 1, dots) and at P = 1.7 GPa (curve 2, circles). Straight dashed lines designate extrapolated *ρ*_*N*_(*T*). The inset (**a**) illustrates the method of determining Т* with the use of the criterion [ρ(Т) − ρ_0_)]/aТ = 1^45^, insert (**b**) - pressure dependence of Т_с_ and ρ(300 К).

Temperature dependences of the resistivity ρ(Т) = ρ_ab_(Т) of the Y_0.95_Pr_0.05_Ba_2_Cu_3_O_7−δ_ single crystal measured at atmospheric pressure (P = 0) (curve 1) and at P = 1.7 GPa (curve 2) are shown in Fig. 1. In this figure, the values of T_c_ and ρ(300) at P = 0 were 85.2 K and 190 µΩ·cm, respectively. Thus, compared to the optimally doped (OD) pure YBaCuO single-crystal samples, the critical temperature after Pr doping dropped by 5–7 K, at the same time being accompanied by the 30–40 µΩ·cm increase in ρ_ab_(300), which is consistent with previous studies^12,25–29^. At all applied pressures, the dependences ρ(T) had the shape typical to OD HTSCs^39,41–44^. In the wide temperature range from T* to 300 K, the ρ(T) dependencies are linear with the slope dρ/dT ≈ 0.61 µΩ·cm/K (P = 0) and dρ/dT ≈ 0.37 µΩ·cm/K (P = 1.7 GPa). The slope was determined by approximating the experimental dependences ρ(T) by the straight line equation ρ(T) = ρ_0_ + aT, where a = dρ/dT and ρ_0_ is the residual resistance which is the intersection of this straight line with the Y axis at T = 0. The approximation confirmed high linearity of the dependencies with the root-mean-square error of 0.002 ± 0.001 in the specified T interval for all the samples studied. The deviation of ρ(T) from linearity towards smaller values determines the PG opening temperature T*. For the sake of accurate determination of T*, the criterion (ρ(T) − ρ_0_)/aT = 1 was used^45^, which is obtained by transforming the equation of the straight line. In this case, T* is defined as the deviation temperature of (ρ(T) − ρ_0_)/aT from 1, as shown at Fig. 1, box (a). In this case, the deviation from linearity is very sharp, which allows T* to be determined with great accuracy. In total five curves obtained at pressures P = 0, 0.45, 0.92, 1.27 and 1.7 GPa, which can be considered as 5 different samples (Y0 – Y5, respectively) were analysed. The corresponding resistive curves for intermediate pressures have a similar shape and are located between the ρ(T) curves at Р = 0 and Р = 1.7 GPa. They are not shown in Fig. 1 for clarity.

The temperature of the resistive transition to the SC state, T_c_ (R = 0) was determined by extrapolation of the linear part of the SC transition towards the intersection with the temperature axis^25–27,38,39^. Notably, the width of resistive transitions ΔT_c_ = T_c_(0.9ρ_n_) − T_c_(0.1ρ_n_), where ρ_n_ is the resistivity of the sample above the transition^38^, in the case of YPrBaCuO single crystal, is rather small. At Р = 0, ΔТ_с_ ≈ 1.4 K and ΔТ_с_ ≈ 2 K at Р = 1.7 GPa, which gives dΔТ_с_/dP ≈ 0.35 K∙GPa^−1^. At the same time, in OD YBCO with T_c_ = 91.07 K, which does not contain defects, the resistive transitions are noticeably narrower, namely, ΔТ_с_ ≈ 0.3 K (P = 0), ΔТ_с_ ≈ 0.5 K (P = 0.95 GPa) and dΔТ_с_/dP ≈ 0.18 K∙GPa^−1^ ^39^. It is significant that in all the cases the pressure increases the resistive transition width. This effect is most pronounced in slightly doped (SD) YBCO single crystals (T_c_(P = 0) = 49.2 K), where d∆T_c_/dP ≈ 0.65 K∙GPa^−1^ ^38^ and especially in HoBCO single crystals (T_c_(P = 0) = 61.3 K, and μ_Ho_ ≈ 10.5μ_B_ and m_eff_ = 9.7μ_*В*_^12^), containing prolonged defects in the form of TB^46^. In the latter case, dΔТ_с_/dP ≈ 3.5 K∙GPa^−1^. Resistive parameters of the samples under study at various pressures are given in Table 1.

**Table 1:** Changes in the parameters of the Y_0.95_Pr_0.05_Ba_2_Cu_3_O_7−δ_ single crystal under pressure.

P (GPa)	ρ(300 K) µΩ(cm)	ρ(100 K) µΩ(cm)	T_c_ (K)	T_c_^mf^ (K)	T_G_ (K)	T_o_ (K)	T_01_ (K)	∆T_fl_ (K)	d_01_ (Å)	ξ_c_(0) (Å)
**0**	190.05	69.22	85.2	85.85	85.97	88.0	92.77	6.8	6.43	1.84
**0.45**	171.84	67.41	86.8	87.51	87.66	90.18	96.72	9.06	6.35	2.03
**0.92**	154.42	65.10	87.6	88.42	88.58	91.32	98.78	10.2	6.3	2.12
**1.27**	142.0	61.84	88.1	88.77	88.92	91.72	99.18	10.26	6.22	2.14
**1.70**	134.0	57.94	89.1	89.17	89.4	92.15	99.53	10.13	6.22	2.16

**Figure 2:**
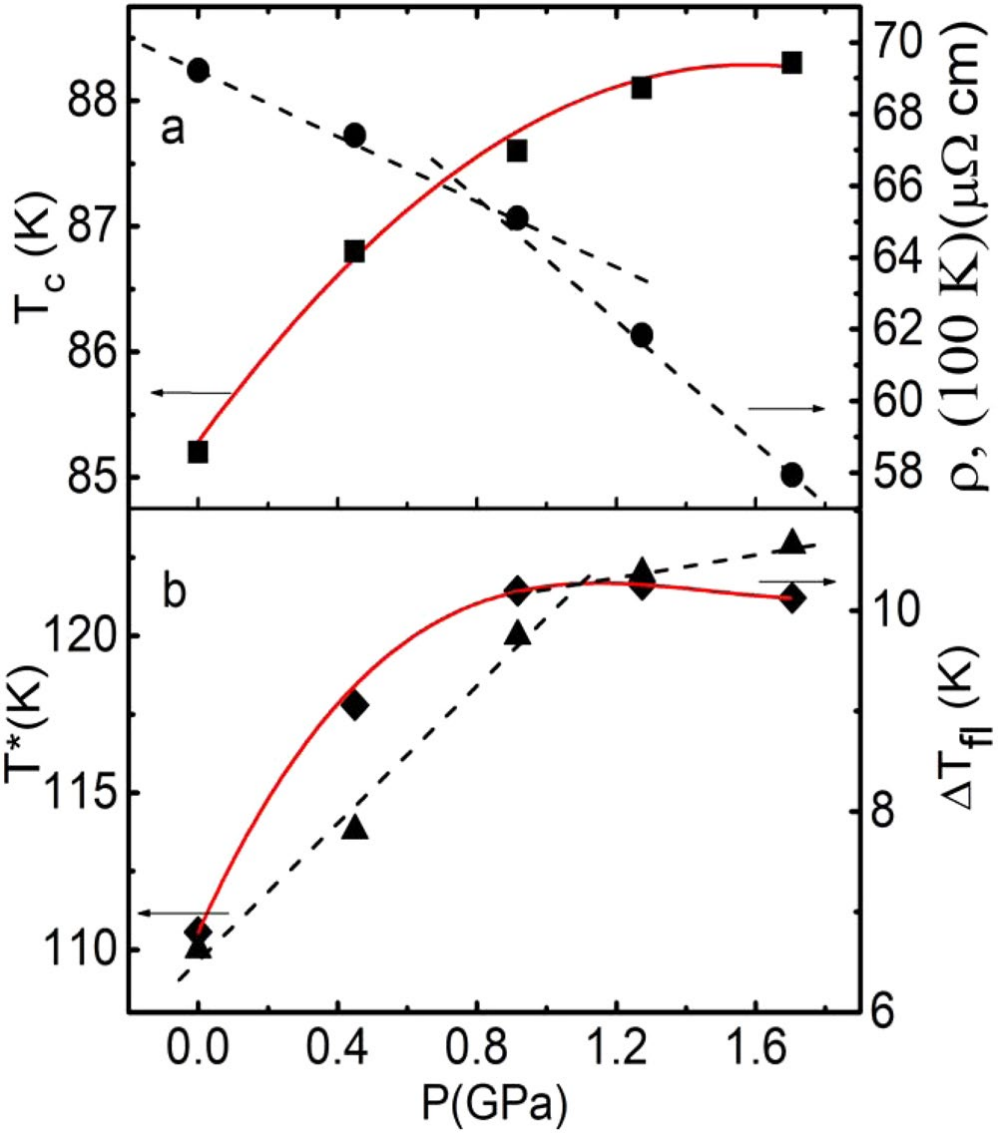
The pressure dependencies: Т_с_ (panel a, squares), Т* (panel b, triangles), ρ(100 K) (panel a, dots) and ΔТ_fl_ (panel b, diamonds) for Y_0.95_Pr_0.05_Ba_2_Cu_3_O_7–δ_ single crystal. The red curves are the 4^th^ degree polynomial approximations. The dashed lines present the linear interpolation.

Just as in the overwhelming majority of cuprates, in the YPrBaCuO single crystal under study, the hydrostatic pressure leads to the increase of T_c_ at a rate dT_c_/dP = +1.82 K∙GPa^−1^ and decreases the resistance as dlnρ/dP = −(10.5 ± 0.2) %∙GPa^−1^ (Fig. 1, inset b, and Fig. 2a). However, there are some noticeable differences. Typically, in cuprates in the pressure range under consideration, the dependences of T_c_, T*, and ρ on P are linear^27,31,47^. It is true not only for YBCO^25,46,48^. In our case, the dependence of T_c_ on P is appreciably nonlinear and already comes to saturation at P ≥ 0.9 GPa (Fig. 1, inset b, and Fig. 2a). At this pressure the dependence ρ(300) on P also deviates from linearity towards higher values (Fig. 1, inset b). However, the most interesting pressure dependences are demonstrated by ρ (100), measured in the pseudogap region. So does the T*(P) (Fig. 2a,b). Both dependences clearly change the slope at Р ~ 0.9 GPa. As it will be shown below, most of the measured parameters of YPrBaCuO demonstrate some peculiarity at P ~ 0.9 GPa.

We also note a very small, compared to pure YBCO single crystals with a similar T_c_ = 85.2 K^44^, value of the PG temperature T* = 110 ± 0.3 K at P = 0. Аny dopants, including Pr, may play the role of impurities in the sample, since their random Coulomb fields create additional scattering centres for charge carriers^16–18^. It can be assumed that additional defects induced by PrBCO, as well as the magnetic moment of PrBCO, prevent the establishment of phase coherence and the formation of fluctuating Cooper pairs (FCPs) above T_c_, consequently reducing T*^18,24^. Another difference is in the relatively weak decrease of resistance under pressure, especially if one considers that the pressure in this case is 1.7 times greater than the pressure we employed in previous studies^38,39,46^. The obtained value dlnρ/dP = - (10.5 ± 0.2) %∙GPa^−1^ is 1.6, and it 1.8 times less than in OD^39^ and SD^38^ pure YBCO single crystals, respectively. One of the possible reasons for the decrease in resistance is the redistribution of charge carriers under pressure from the CuO chains into the CuO_2_ planes, which should lead to an increase in the density of charge carriers, n_f_, in the planes^12^. As noted above, in YPrBCO part of the charge carriers is localized, which can suppress the rise of n_f_ under pressure in the CuO_2_ planes and lead to the observed decrease in the rate of ρ reduction. The observed unexpected increase in the PG temperature to T* = 122.9 ± 0.3 K at P = 1.7 GPa is discussed below.

In accordance with the phase diagram of cuprates^7–9,41^, an increase in n_f_ in the CuO_2_ planes under pressure should lead to an increase in the T_c_ of the samples (see review^12^ and references therein). In the single crystal YPrBaCuO under study we have dT_c_/dP = +1.82 K∙GPa^−1^ (Fig. 1, inset b). This is ~2.7 times less than in SD YBCO single crystals, where dT_c_/dP = +5 K GPa^−1^ ^38^, but ~2.5 times higher than in OD of YBCO single crystals, where the increment rate of the critical temperature dT_c_/dP = +0.73 K∙GPa^−1^ ^39^. The dT_c_/dP observed in YPrBCO is approximately the same magnitude as it should be in pure YBCO with T_c_ ≈ 85 K. Thus, the localization of charge carriers due to the presence of PrBCO in this case has little effect on T_c_. This result once again confirms that the mechanisms of the effect of hydrostatic pressure on the critical temperature and the resistivity of both YPrBCO and YBCO single crystals, are most likely different^12,46^.

While determining dТ_с_/dP, two effects should be distinguished, the first one associated with change in electron-phonon interaction, lattice parameters, bonding in-between layers, etc. (true pressure effect), and the second effect of change in n_f_ due to redistribution of labile oxygen (pressure relaxation effect)^12,25–31^. The dependence of T_c_ on pressure can be represented by the formula^25,49^.

1 $$\frac{d{T}_{c}}{dP}={(\frac{\partial {T}_{c}}{\partial P})}_{n}+{(\frac{\partial {T}_{c}}{\partial n})}_{P}\cdot \frac{dn}{dP}$$

where n = n_f_ is the density of charge carriers in the sample. Thus, the first term on the right of Eq. (1) characterizes the true direct effect of pressure, and the second is the result of the change in n_f_ under pressure. As was shown above, in YPrBCO, the change in n_f_ is relatively small due to the possible localization of charge carriers. Therefore, it is the true pressure effect that should be responsible for dT_c_/dP in YPrBCO. Various theoretical models for describing the behaviour of dT_c_/dP in cuprates are discussed in detail previously^12^. The small dT_c_/dP values in the OD samples^39,47^ and the noticeable effect of pressure on the T_c_ value in the SD YBCO single crystals^38,47^, observed in the experiment, can be explained in the framework of the model assuming the presence of a Van Hove singularity in the spectrum of charge carriers^50,51^, which is characteristic to lattices with strong bonding. In OD crystals (with T_c_ ~ 90 K) the Fermi level is in the valley formed between the two peaks of the density of states. Importantly, the density of states at the Fermi level N (E_F_) depends upon the orthorhombic distortion (a–b)/a^50^. It should be stressed that under hydrostatic pressure, the variation of the (a–b)/a ratio is small (it is determined only by the difference in compression modules along the a and b axes). Therefore, the change in T_c_ under hydrostatic pressure is relatively small.

For SD crystals with low T_c_ ~ 60 K, the Fermi level can be shifted from the middle of the zone (among other factors due to doping with substitutional elements^52,53^) and is located away from the peak of the density of states. Therefore, if the critical temperature value is primarily determined by the density of electronic states, then a shift of the Fermi level towards the peak of the density of states, when the hydrostatic pressure is applied, can provoke a significant increase in the absolute value of dТ_с_/dP^25,38,47^. Accordingly, the crystals with intermediate values of T_c_, including weakly Pr doped YPrBaCuO, should demonstrate intermediate values of dT_c_/dP, as it is observed experimentally.

### Fluctuation conductivity

Fluctuation conductivity (FLC) at all applied pressures was determined from the analysis of the excess conductivity σ′(T), which was calculated by the following equation as the difference between the measured resistivity ρ(T) and the linear normal-state resistivity of the sample ρ_N_(T) = аT + ρ_0_ extrapolated to low temperatures^39–42,54^:

2 $$\begin{array}{c}{\rm{\sigma }}^{\prime} ({\rm{T}})={\rm{\sigma }}({\rm{T}})-{{\rm{\sigma }}}_{{\rm{N}}}({\rm{T}})=[1/\rho ({\rm{T}})]-[1/{{\rm{\rho }}}_{{\rm{N}}}({\rm{T}})],\,{\rm{or}}\\ \,{\rm{\sigma }}^{\prime} ({\rm{T}})=[{{\rm{\rho }}}_{{\rm{N}}}({\rm{T}})-{\rm{\rho }}({\rm{T}})]/[{\rm{\rho }}({\rm{T}}){{\rm{\rho }}}_{{\rm{N}}}({\rm{T}})].\end{array}$$

As shown in previous studies^42–44,55^, the linear temperature dependence of the resistivity in the high temperature region is a distinctive feature of the normal state of cuprate HTSCs, for which stability of the Fermi surface takes place. Below the opening temperature of the pseudogap T* the Fermi surface may undergo rearrangement^7,9,55–57^. As a result, at T ≤ T*, not only practically all properties of HTSCs change and ρ(T) deviates from the linear dependence^57^ but also the density of states at the Fermi level begins to decrease^58,59^, which by definition is called a pseudogap^5–9,60^. Obviously, the resulting excess conductivity σ′(T), defined by Eq. (2), should contain information on the temperature dependence of both the FLC and PG^38,39,54,61^. This approach was used to analyze σ′(T) at all applied pressures. We consider below in more detail the procedure for determining the FLC and PG in the model of local pairs (LPs)^3–6,44,60^ on the example of the samples Y0 (P = 0) and Y5 (P = 1.7 GPa).

Prior to beginning of the analysis within the framework of the LPs – model it is necessary to determine the critical temperature in the mean field approximation T_c_^mf^, which separates the FLC region from the region of critical fluctuations^12,60,62^, i.e. the fluctuations of the SC order parameter Δ immediately near T_c_ (where Δ < kT), ignored in the Ginzburg-Landau theory^63^. The T_c_^mf^ is an important parameter of both FLC and PG – analysis, because it determines the reduced temperature

3 $${{\rm{\varepsilon }}=({\rm{T}}/{\rm{T}}}_{{\rm{c}}}^{{\rm{mf}}}-1)$$

which is present in all equations of this article. In HTSCs near T_c_, the coherence length along the c axis, ${\xi }_{c}(T)={\xi }_{c}(0){(T/{{T}_{c}}^{mf}-1)}^{-1/2}$ is greater than the corresponding unit cell size of YPrBCO d = c = 11.7 Å^64^, and the FCPs interact in the entire volume of the superconductor. Accordingly, this is the area of 3D fluctuations. As a result, up to the temperature of the 3D – 2D crossover T_0_ > T_c_^mf^, the σ′(ε) is always extrapolated by the fluctuation contribution of the Aslamazov – Larkin (AL) theory^65^ for 3D systems^42,44,54,60^.

4 $${\sigma ^{\prime} }_{AL3D}(T)={C}_{3D}\frac{{e}^{2}}{32\hslash {\xi }_{c}(0)}{\varepsilon }^{-\frac{1}{2}}$$

**Figure 3:**
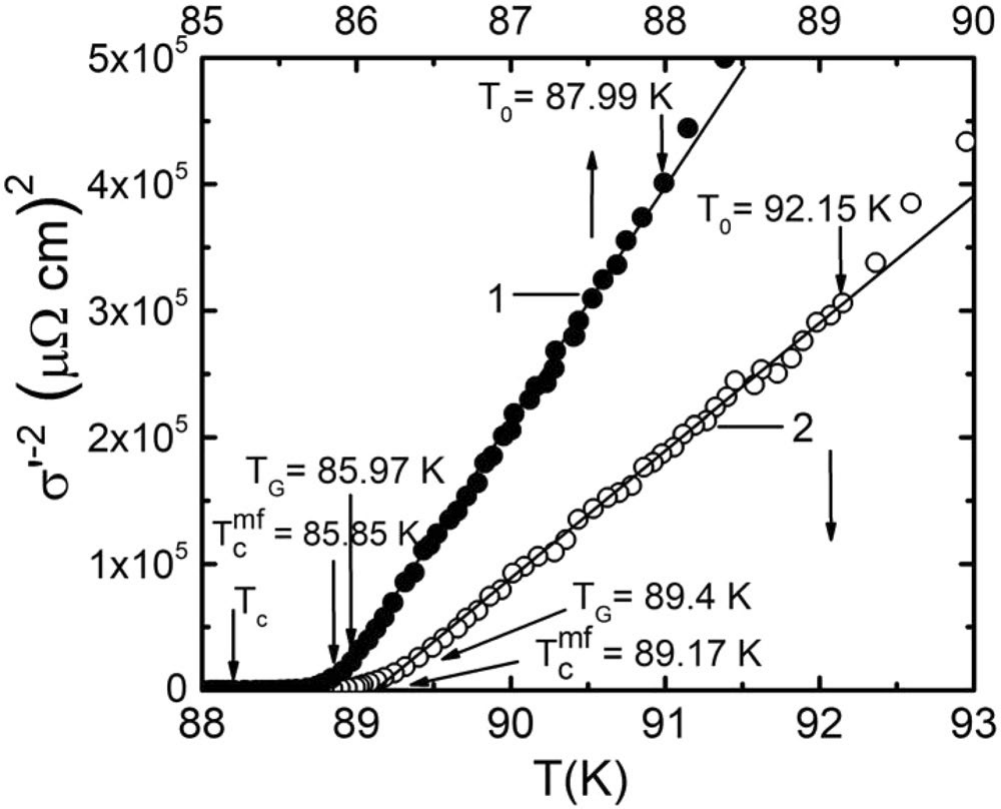
Temperature dependencies σ^′−2^(Т) for Y_0.95_Pr_0.05_Ba_2_Cu_3_O_7−δ_ single crystal at P = 0 (curve 1, points) and P = 1.7 GPa (curve 2, circles) that define T_c_^mf^. The arrows point to the characteristic temperatures (refer to the text). The straight lines are to guide the eye.

From this formula it is easy to obtain that σ^′−2^(T) ~ ε ~ T − T_c_^mf^. Obviously, the extrapolated linear dependence σ^′−2^ (T) turns to 0 just at T = T_c_^mf^ (Fig. 3)^62^. In addition to T_c_^mf^ and T_c_, Fig. 3. also shows the Ginzburg temperature T_G_, down to which the mean-field theories operate with decreasing T^27,63,66^, and also the temperature of the 3D–2D crossover T_0_, which limits the 3D – AL region of fluctuations from the top^27,44,60^.

The significant difference between the results shown in Fig. 3, from the analogous dependences obtained on pure YBCO single crystals^12,38,39^, is in the deviation of the experimental data leftwards and upwards from the linear dependence of σ′^−2^(T) above T_0_. Such σ′^−2^(T) dependence indicates the absence of the fluctuation contribution of the Maki – Thompson (MT)^67^ in the FLC and is typical for samples with defects^62,68^. In well-structured films^12,44,60^ and single crystals of YBCO^38,39^ the MT fluctuation contribution is always observed, and the experimental points above T_0_ deviate to the right from the linear dependence σ′^−2^(T). The result confirms the above conclusion about the presence of additional defects in the sample induced by PrBCO^18,24,35,36^. The noticeable scatter of the experimental points in the temperature range T_0_ − T_G_ leads to a specific dependence of PG ∆*(T) in the specified temperature range, which will be discussed in what follows. Additionally, under pressure, the slope of the experimental curves noticeably changes (shown by the straight lines in Fig. 3). It is significant that the slope begins to change visibly only at Р ≥ 0.9 GPa, indicating a non-monotonic increase in σ′(T) with increasing pressure. We emphasize that we did not find a change in the slope of the σ′^−2^(T) dependencies neither in the SD^38^ nor in the OD^39^ YBCO single crystals, however, it should be noted that the maximum value of P in these studies did not exceed 1 GPa.

**Figure 4:**
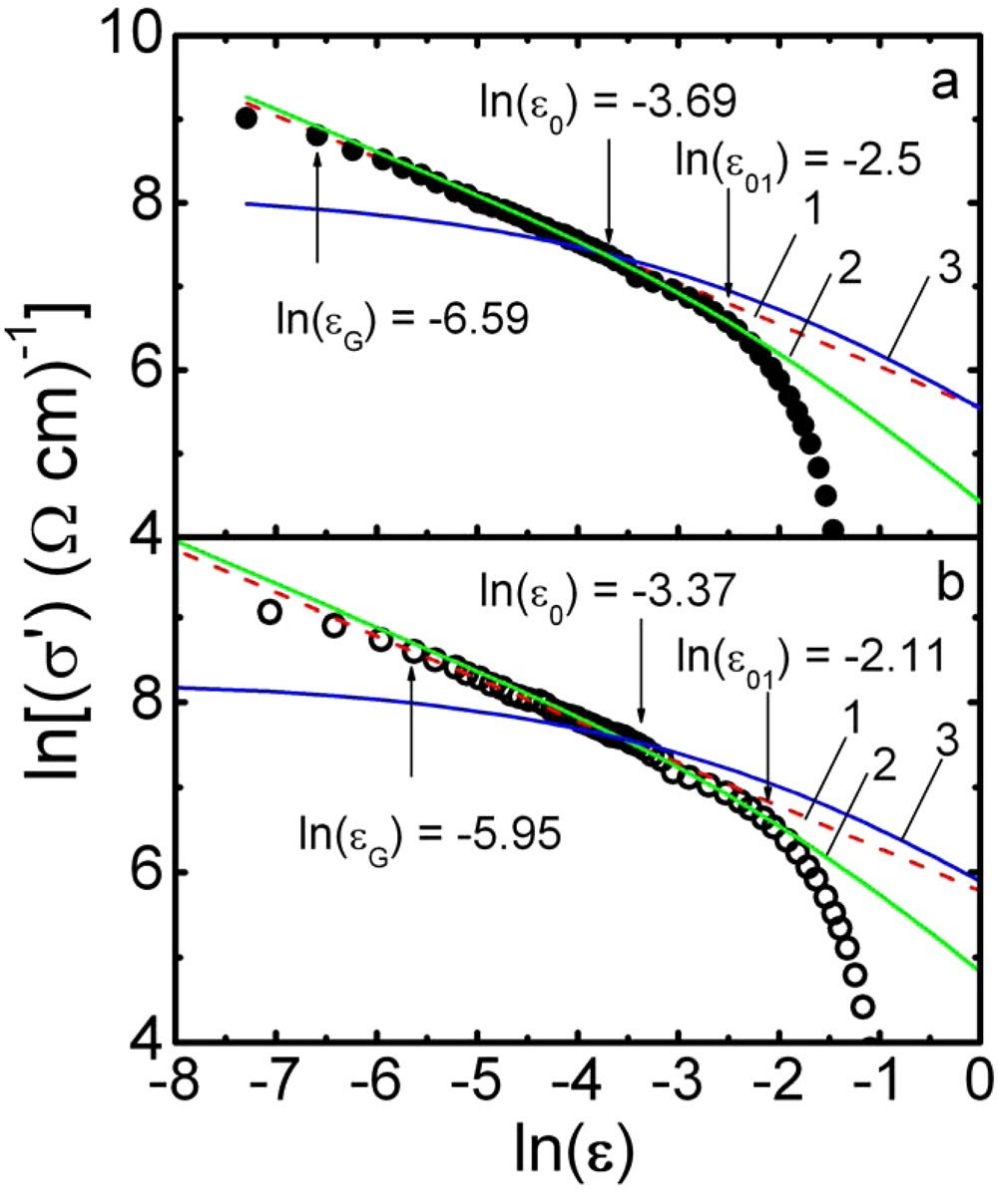
Dependence of lnσ′ on lnε of Y_0.95_Pr_0.05_Ba_2_Cu_3_O_7−δ_ single crystal for Р = 0 (panel a, dots) and P = 1.7 GPa (panel b, circles) in comparison with fluctuation theories: АL – 3D (red dashed line 1), LD (green solid curve 2) and МТ – 2D (blue solid curve 3).

Having determined T_c_^mf^, we find ε. After that we can clarify the role of fluctuating pairing in the formation of PG^3–8,60–62^. To do this, we construct the dependence lnσ′ vs lnε, as shown in Fig. 4 for samples Y0 (Р = 0) and Y5 (Р = 1.7 GPa). As expected, regardless of the presence of pressure, near T_c_ the FLC is perfectly approximated by the fluctuation contribution of AL for 3D systems (Eq. 4). In the logarithmic coordinates, the latter is plotted as dashed red straight lines (1) at Fig. 4 with slope λ = −1/2. This result confirms the above conclusion that classical 3D – AL FLC is always realized in cuprate HTSCs with ξ_c_(T) > d when T tends to T_c_^12,27,41–44,69,70^. The linear dependence lnσ′ (lnε) is maintained up to the temperature T_0_ = 88.0 K (lnε_0_ = −3.69, P = 0), at which the 3D-2D crossover occurs^67,71^, and the experimental points deviate towards smaller values. At Т = Т_0_, ξ_с_(Т_0_) = d = 11.67 Å^44,60^. In accordance with previous studies^12,44,60^ we obtain:

5 $${{\rm{\xi }}}_{{\rm{c}}}(0)={\rm{d}}\sqrt{{{\rm{\varepsilon }}}_{0}}$$

After getting ε_0_ from the Eq. (5) we find ξ_c_(0) = (1.84 ± 0.02) Å for Р = 0. Similarly, the values of ξ_с_(0) are obtained at all other pressures (Table 1). These values of ξ_с_(0) are in reasonable agreement with the data reported for YBCO^27,38,39,72,73^, and at Р = 0 they actually coincide with ξ_с_(0) = (1.86 ± 0.02) Å, found for the two-layer film YBCO-PrBCO with the similar T_c_ = 85 K (sample SD1)^23^.

Above T_0_ (Fig. 4) we have ξ_с_(T) < d, and the sample loses its 3D state^42,44,67,71^. However, as before, in the temperature range T_0_ < T < T_01_ (lnε_0_ < lnε < lnε_01_, Fig. 4) ξ_c_(T) > d_01_ where d_01_ is the distance between the inner conducting CuO_2_ planes, and the CuO_2_ planes are connected by the Josephson interaction^60,67,71^. As a result, two-dimensional (2D) FLC is realized in HTSC. However, in contrast to well-structured YBCO single crystals^30,38,39^, in this case the temperature dependence of the FLC is described by the Lawrence – Doniach (LD) model (solid green curves 2 at Fig. 4)

6 $${\sigma ^{\prime} }_{LD}={C}_{LD}\frac{{e}^{2}}{16\hslash d\sqrt{1+2\alpha }}{\varepsilon }^{-1}$$

which is a special case of the Hikami – Larkin (HL) theory^67^. Here $\alpha =2{[{\xi }_{c}(0)/d]}^{2}{\varepsilon }^{-1}$ is the coupling parameter. Near T_c_, where ξ_c_(T) ≫ d, Eq. (6) is transformed into a 3D – AL (Eq. 4). Such temperature dependence σ′(ε) is typical for HTSC samples with defects^62,68^ arising under the influence of PrBCO. Accordingly, the fluctuation 2D-MT contribution to σ′(ε) is

7 $${\sigma ^{\prime} }_{MT2D}={C}_{2D}\frac{{e}^{2}}{8d\hslash }\cdot \frac{1}{1-\alpha /\delta }\cdot \,\mathrm{ln}((\delta /\alpha )\cdot \frac{1+\alpha +\sqrt{1+2\alpha }}{1+\delta +\sqrt{1+2\delta }}))\cdot {\varepsilon }^{-1}$$

calculated for HTSCs in the HL theory^67^. It is characteristic to well-structured samples^12,38,39,44^ and is completely suppressed in the given case. The MT contribution is determined by the pair-breaking processes in a sample in absence of the defects, i.e. it depends on the lifetime of the FCPs τ_φ_^67^:

8 $${\tau }_{\varphi }\beta T=\pi \hslash /8{k}_{B}\varepsilon =A/\varepsilon $$

where A = 2.998 ∙ 10^−12^ sK and

9 $$\delta =\beta \frac{16}{\pi \hslash }{(\frac{{\xi }_{c}(0)}{d})}^{2}{k}_{B}T{\tau }_{\varphi }$$

is the pair-breaking parameter. The factor β = 1.203 (*l*/ξ_ab_) (where *l* is the mean free path, ξ_ab_ is the coherence length in the ab-plane) corresponds to the case of the clean limit (*l* > ξ_ab_), which is always realized in HTSCs (see^12,60^ and references therein). Curves (3), calculated using Eq. (7) with d = 11.67 Å and the values ξ_c_(0) determined from the experiment (Table 1), along with τ_φ_ (100 K) β = 11.9 · 10^−13^ s (P = 0), τ_φ_ (100 K) β = 8.9 · 10^–13^ s (P = 1.7 GPa), are also shown in Fig. 4 and, as expected, do not match the experiment.

Above T_01_ [i.e. corresponding to lnε_01_ at Fig. 4 (T_01_ = 92.77 K and lnε_01_ = −2.5, P = 0)], the experimental data deviate from the LD curve towards smaller values. Thus, classical fluctuation theories^65,67^, based on the concept of the existence of incoherent FCPs in cuprate HTSCs at T > T_c_^5,6,70^, successfully describe the excess conductivity σ′(*Т*) only up to the temperature T_01_. Above T_01_ we have ξ_c_(T) < d_01_^12,23,60^ and disappearance of the Josephson interaction between the inner conducting planes of CuO_2_. In this case, both superconducting and normal charge carriers are confined directly in the CuO_2_ planes, which are now not interconnected by any correlation interaction^67,71^. For this reason, above T_01_ the fluctuation theories do not describe the experiment, as it is clearly seen from the results shown in Fig. 4. It is obvious that *ξ*_*c*_(*T*_01_) = *ξ*_*c*_(0)${\varepsilon }_{01}^{1/2}$ = *d*_01_. Since ξ_c_ (0) is determined by the temperature of the 3D–2D crossover T_0_ (Eq. 5), then the condition *ξ*_*c*_(0) = $d\sqrt{{\varepsilon }_{0}}={d}_{01}\sqrt{{\varepsilon }_{01}}$ = (1.84 ± 0.02) Å (P = 0) must be fulfilled. Taking as noted above, d = c = 11.67 Å, for P = 0 we get ${d}_{01}=d\sqrt{\varepsilon /{\varepsilon }_{01}}=(6.43\pm 0.05)$ Å and, respectively, *d*_01_ = (6.22 ± 0.05) Å for P = 1.7 GPa. Similarly, d_01_ values were obtained for all other pressures (refer to Table 1). Thus, the pressure somewhat reduces the inter-plane distance in YPrBCO single crystals (refer to Fig. 4), which is reasonable^31,37^ given that the pressure noticeably reduces all the parameters of the YBCO unit cell^74^.

The fact that in the temperature range ΔT_fl_ = T_01_ − T_G_ FLC obeys the classical fluctuation theories means that T_01_ is the temperature up to which the order-parameter phase stiffness, as well as the superfluid density n_s_, have to maintain in HTSCs^5,6^.

This is confirmed by the experiment^75–78^. Consequently, in this temperature range, the FCPs largely behave like the SC but non-coherent pairs (the so-called “short-range phase correlations”^4–6,23,41,70^), as noted above. Therefore, the problem of the temperature T_01_ is very important. In some cases, e.g., ΔT_fl_ = 15.6 K and T_01_ ~ 16 K is higher than T_c_. This result, T_01_/T_c_ ≈ 1.19, was obtained on a well-structured two-layer YBCO-PrBCO film with T_c_ = 88.5 K (sample SD2)^23^, in which independently deposited PrBCO layers do not create additional defects in the YBCO layers^19,23^. The similar film with T_c_ = 85 K (sample SD1 in^23^) is characterized by ΔT_fl_ = 11.4 K and T_01_/T_c_ ≈ 1.13. In the YPrBCO single crystal under study with practically the same T_c_ = 85.2 K, the ΔT_fl_ = 6.8 K and T_01_/T_c_ ≈ 1.05 at P = 0, i.e., it is 1.7 times less than in the well-structured SD1 film. This result once again confirms the conclusion that it is the defects induced by magnetic impurities in the form of PrBCO inclusions, arising in YBCO doped with Pr, which prevent the establishment of phase coherence of the FCPs and noticeably reduce the region of SC fluctuations. Under the pressure of 1.7 GPa, the critical temperature of the YPrBCO single crystal increases to T_c_ = 89.1 K becoming almost the same as in the SD2 film. Correspondingly, the range of SС fluctuations ΔT_fl_ = 10.13 K as well as T_01_/T_c_ ≈ 1.11, i.e. increase noticeably (refer to Table 1). At the same time the C-factor C_3D_ also increases, from 0.62 (P = 0) to 0.93 (P = 1.7 GPa), i.e., the better the sample structure, the closer C_3D_ to unity^46,60^. Thus, it can be assumed that the pressure not only decreases d_01_, but also reduces the number of defects in the sample, minimizing the degree of disorder, consistently with previous studies^28,74^.

The increase in ΔT_fl_ also occurs non-monotonously. Up to Р = 0.92 GPa, the range of SC fluctuations rapidly widens, and then comes to saturation (Fig. 2b, red curve). From Fig. 2. it can be seen that the dependence ΔT_fl_ (P) is essentially the same as the dependence T_c_ (P). Taking into account the observed decrease in ρ(P) (Fig. 2a) it can be assumed that the reason for the increase in both T_c_ and ΔT_fl_ is in growth of the density of charge carriers in the sample under applied P. For the first time such non-monotonic behaviour of all the functions (Fig. 2) was found. It can be attributed to specific features of the Y_0.95_Pr_0.05_Ba_2_Cu_3_O_7−δ_ single crystal under study.

### Temperature dependence of the pseudogap

In the model of local pairs (LPs)^3–6,54,60,75–78^, it is assumed that the deviation of ρ(T) from the linear dependence is due to opening of the PG at T* ≫ T_c_, leading to appearance of the excess conductivity σ′(T), Eq. (1), as a result of formation of the LPs. This in turn means that the excess conductivity σ′(T) arising from such processes should contain information about the magnitude and temperature dependence of PG.

To obtain such information, one needs to have an equation that describes the experimental dependence of σ′(T) over the entire temperature range from T* to T_c_ including the pseudogap parameter Δ*(T) in an explicit form. In view of the absence of a rigorous theory, the corresponding formula for σ′(T) was previously proposed^61^

10 $$\sigma ^{\prime} (\varepsilon )=\frac{{e}^{2}{A}_{4}(1-\frac{T}{{T}^{\ast }})(\exp (-\frac{{\Delta }^{\ast }}{T}))}{16\hslash {\xi }_{c}(0)\sqrt{2{\varepsilon }_{c0}^{\ast }\,\sinh (2\varepsilon /{\varepsilon }_{c0}^{\ast })}}$$

where (1 − T/T*) determines the number of pairs appearing at T ≤ T*, and exp (−∆*/T) gives the number of pairs destroyed by thermal fluctuations below T_pair_. Solving Eq. (10) for ∆*(T), we obtain the equation for PG

11 $${\Delta }^{\ast }(T)=T\,\mathrm{ln}\,\frac{{e}^{2}{A}_{4}(1-\frac{T}{{T}^{\ast }})}{\sigma ^{\prime} (T)16\hslash {\xi }_{c}(0)\sqrt{2{\varepsilon }_{c0}^{\ast }\,\sinh (2\varepsilon /{\varepsilon }_{c0}^{\ast })}},$$

where σ′(T) is the excess conductivity experimentally determined.

**Figure 5:**
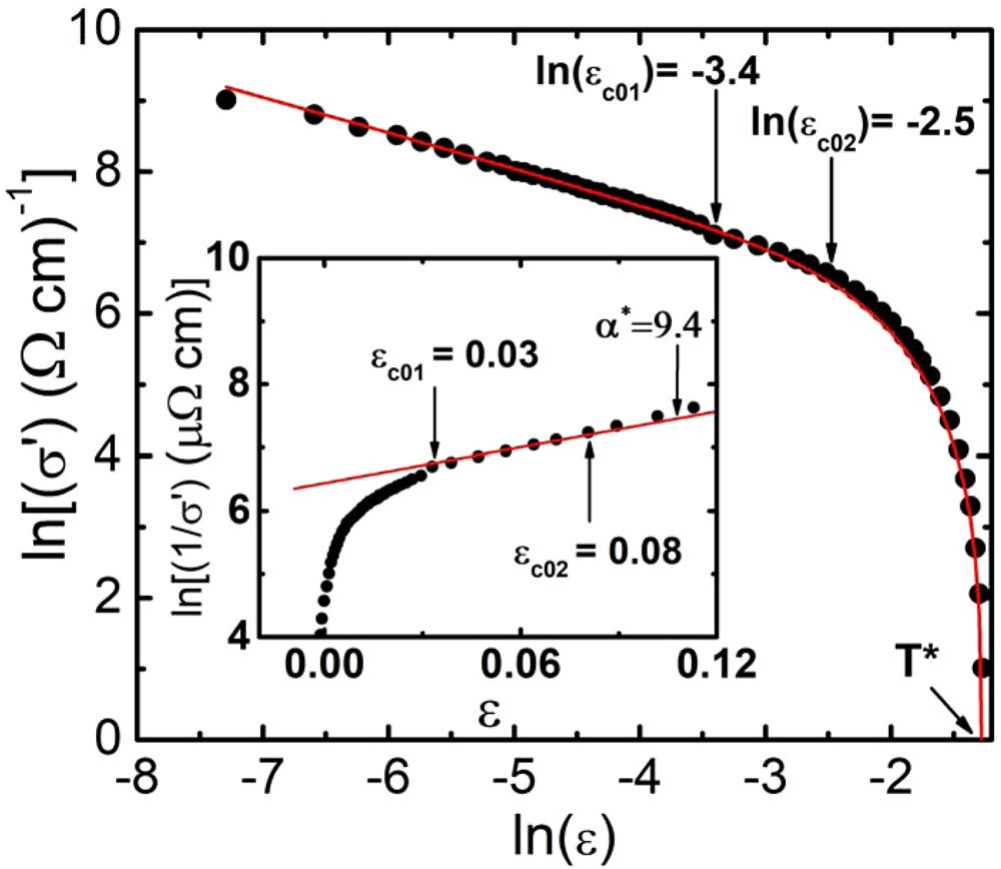
Dependence of ln(σ′) vs ln(ε) (points) of the Y_0.95_Pr_0.05_Ba_2_Cu_3_O_7−δ_ single crystal for P = 0, plotted over the entire temperature range from T* to T_c_^mf^. The red curve is the approximation of the experimental data by Eq. (10). Inset: ln(σ^′–1^) as a function of ε. The red line marks the linear part of the curve, the inverse slope of which is 1/α* = ${\varepsilon }_{c0}^{\ast }$ = 0.11.

In addition to T_c_^mf^, T*, ξ_c_(0) and ε, that are already defined above, Eqs. 10 and 11 contain the coefficient A_4_, which has the same meaning as the C-factor in the theory of FLC, and also ∆* along with the theoretical parameter ${\varepsilon }_{c0}^{\ast }$^72,73^, which determines the shape of theoretical curves for T > T_01_^60,61^. Within the framework of the LPs model, all these parameters are also directly determined from experiment^23,24,38,39,61^. In order to find ${\varepsilon }_{c0}^{\ast }$, we use the experimental fact that in the interval lnε_01_ < lnε < lnε_02_ (lnε_01_ = −3.4; lnε_02_ = −2.5, at Fig. 5) the excess conductivity σ^′−1^ ~ exp (ε), which is apparently an intrinsic property of cuprates^23,61,72,73^. Accordingly, in the temperature range ε_с01_ < ε < ε_с02_ (89.2 < T < 92.8 K) (see inset to Fig. 5), ln (σ′^−1^) is a linear function of ε with the slope α^*^ = 9.4, which defines the parameter ${\varepsilon }_{c0}^{\ast }$ = 1/α^*^ = 0.11 at Р = 0^72,73^. The similar graphs with α^*^ decreasing to 5.9, which gives ${\varepsilon }_{c0}^{\ast }$ ≈ 0.17 at P = 1.7 GPa, were plotted for all other pressure values. The latter allowed us to obtain the reasonable values of ${\varepsilon }_{c0}^{\ast }$, which tend to increase with P, as can be seen from Table 2.

**Table 2:** Transformation of the parameters used in the analysis of the PG behaviour in Y_0.95_Pr_0.05_Ba_2_Cu_3_O_7−δ_ single crystal under pressure.

P (GPa)	T* (K)	α	${{\boldsymbol{\varepsilon }}}_{{\bf{c}}{\bf{0}}}^{{\boldsymbol{\ast }}}$	T_pair_ (K)	D*	Gi	∆*(T_G_) (K)
**0**	110	9.4	0.11	108	5	0.0014	216.13
**0.45**	113.8	6.5	0.15	109.7	5	0.0013	218.16
**0.92**	120	6.2	0.16	110.6	5	0.0015	220.83
**1.27**	122	5.5	0.18	114.6	6.0	0.0017	268.04
**1.70**	122.9	5.9	0.17	115	6.4	0.0026	290.02

**Figure 6:**
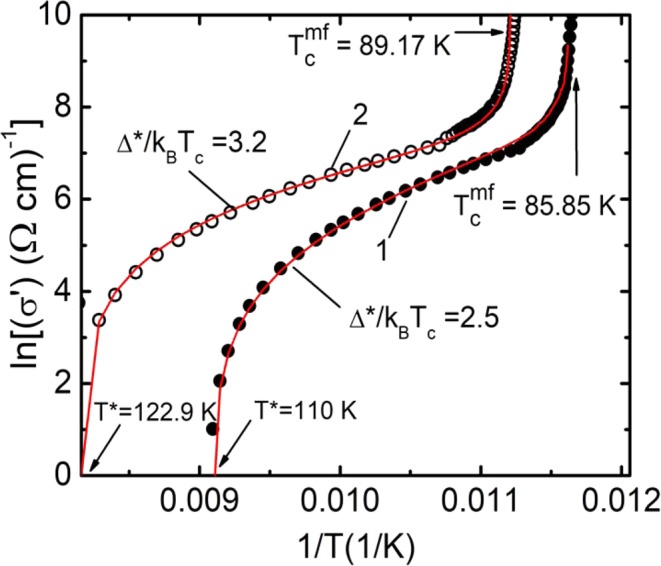
lnσ′ as a function of 1/T for a Y_0.95_Pr_0.05_Ba_2_Cu_3_O_7−δ_ single crystal over the entire temperature range from T^*^ to T_c_^mf^ at P = 0 (curve 1, points) and P = 1.7 GPa (curve 2, circles). Red curves - approximation of the data by Eq. 10 with ∆*(T_G_) = 2.5 K (P = 0) and ∆*(T_G_) = 3.2 K (P = 1.7 GPa).

To find A_4_, using Eq. 10, it is necessary to calculate the dependence σ′(T) and, selecting A_4_, combine with the experiment in the region of 3D – AL fluctuations (solid red curve at Fig. 5), where lnσ′ is a linear function of lnε with the slope λ = −1/2^42,44,60^. However, ∆* still remains undetermined. While using Eq. 10 we take into account the results of works^77,78^ and assume ∆* equal to ∆*(T_G_) = Δ(0), where Δ(0) is the SC gap at T = 0, as noted above. Accordingly, the following relation should be satisfied: D* = 2∆*(T_G_)/k_B_T_c_ = 2Δ(0)/k_B_T_c_^12,23,60^. Ultimately, to estimate ∆*(T_G_), which is used in Eq. 10 we plot lnσ′ as a function of 1/T^23,54,61^. The results for P = 0 and P = 1.7 GPa are shown at Fig. 6. In this case, the shape of the theoretical curve turns out to be very sensitive to the value of ∆*(T_G_). The best approximation for P = 0 is achieved at ∆*(T_G_) = 2.5 k_B_T_c_, i.e. D* = 5.0 ± 0.1 (curve 1 at Fig. 6), which is the typical value for the d-wave superconductors in the strong coupling limit at ambient pressure^12,61,79,80^.

This result seems reasonable, given that the sample is OD in oxygen, with a high T_c_ = 85.2 K even in the presence of Pr. For Р = 1.7 GPa, respectively, we obtain ∆*(T_G_) = 3.2 k_B_T_c_ or D* = 6.4 ± 0.1 (curve 2 in Fig. 6). It can be seen that for the chosen values of the parameters the calculated curves perfectly describe the experiment, including the data at Fig. 5, thus confirming the validity of the present approach. Similar graphs were obtained for all the pressure values chosen, which made it possible to obtain reliable ∆*(T_G_) and D* values for all the samples (refer to Table 2). It is also seen from the figure that the pressure significantly increases the value of the excess conductivity σ′, especially in the high-temperature region. However, it is necessary to point out the threshold nature of the results obtained. Namely, the change in the value of the parameter D* derived from the experimental curves begins only above P ~ 0.9 GPa (see Table 2).

**Figure 7:**
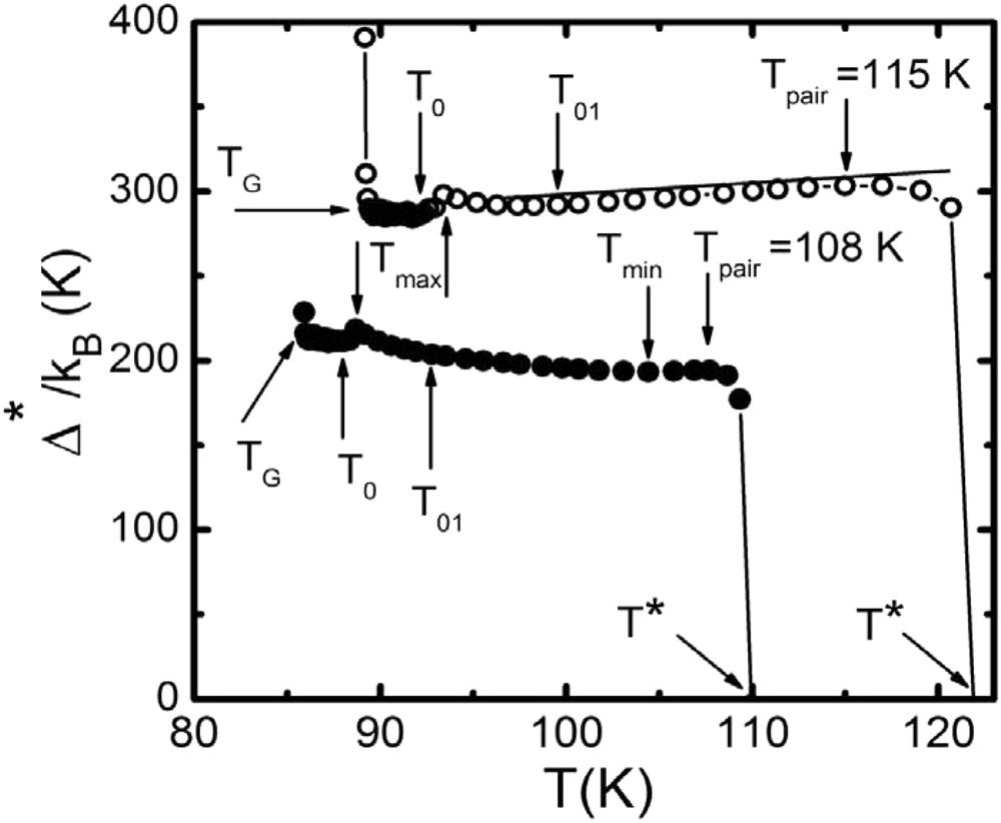
Temperature dependences of the pseudogap ∆*(T) of Y_0.95_Pr_0.05_Ba_2_Cu_3_O_7−δ_ single crystal, calculated from Eq. (11), at Р = 0 (points) Р = 1.7 GPa (circles). The arrows indicate the characteristic temperatures T_pair_, T_min_, T_max_, T_01_, T_0_ and T_G_. The solid line is to guide the eye.

Since all the necessary parameters have been found, we can construct the dependences ∆*(T) for all pressure values. The fact that Eq. 10 perfectly approximates the experimental data (Figs. 5 and 6) allows us to conclude that calculated by Eq. 11 dependences ∆*(T) will gain the correct values and temperature dependencies of PG. The function ∆*(T) for P = 0 based on the experimentally determined parameters T* = 110 K, T_c_^mf^ = 85.85 K, ξ_c_(0) = 1.84 Å, ${\varepsilon }_{c0}^{\ast }$ = 0.11 and A_4_ = 34 is shown at Fig. 7 as solid dots. Accordingly, ∆*(T) for P = 1.7 GPa is plotted at

Fig. 7 in empty circles. This curve was based on the parameters T* = 122.9 K, T_c_^mf^ = 89.17 K, ξ_c_(0) = 2.16 Å, ${\varepsilon }_{c0}^{\ast }$ = 0.17, A_4_ = 85. Similar dependences ∆*(T) for P = 0.45, 0.92 and 1.27 GPa based on the parameter sets given in Tables 1 and 2 are located between these two curves but are not shown here for the sake of not complicating the figure. The pressure noticeably increases the ∆*(T) (Fig. 7). Using the data of Table 2 we find that under the pressure ∆* and D* increase as dln∆*/dP = 0.17 GPa^–1^. We emphasize that for making dln∆*/dP estimates we took the values of ∆*(T_G_), which, as noted above, can be considered as an analogue of the SC gap Δ(0)^12,77,78^. Thus, the hydrostatic pressure increases both ∆* and respectively D*, which is consistent with the results of^31,38,39,81^, where an increase under pressure in both PG and SC gap Δ, as well as increase of the BCS relationship 2Δ(0)/k_B_T_c_, is reported.

However, the values of the derivatives dln∆*/dP = 0.17 GPa^–1^ for the samples with Pr doping are almost two times less than dln∆*/dP = 0.32 GPa^–1^ and dln∆*/dP = 0.36 GPa^–1^ measured for the OD and SD YBCO single crystals^38,39^, respectively. In accordance with previous studies^12,38,39,46,60,81^, the experimentally observed increase in T_c_ under pressure should lead to an increase in both the SC gap Δ and the PG ∆*. Interestingly, in pure OD single crystals of YBCO the T_c_ increases only by 0.7 K, while dln∆*/dP = 0.32 GPa^–1^. In the YPrBCO single crystal under study T_c_ increases by 3 K, and dln∆*/dP = 0.17 GPa^–1^. Thereby there is no direct correlation between the growth of T_c_ and the value dln∆*/dP in this case. As already mentioned, under the hydrostatic pressure the increase in T_c_ may occur due to rising of the density of charge carriers n_f_ in the CuO_2_ planes^12,25–31^ and due to the convergence of the peaks of the density of states^52,53^. Taking into account the results obtained, it is possible to conclude that in YPrBCO the influence of the both given processes on dln∆*/dP is relatively weak due to possible partial localization of the charge carriers^18,19,36^, presence of defects and intrinsic magnetism of PrBCO^21–23^. Another possible mechanism responsible for the increase in the SC gap and the PG is associated with a shift towards lower frequencies of the phonon spectrum of a superconductor under pressure^81^. However, how this mechanism works in presence of the magnetic PrBCO is not clear, and this question remains open.

**Figure 8:**
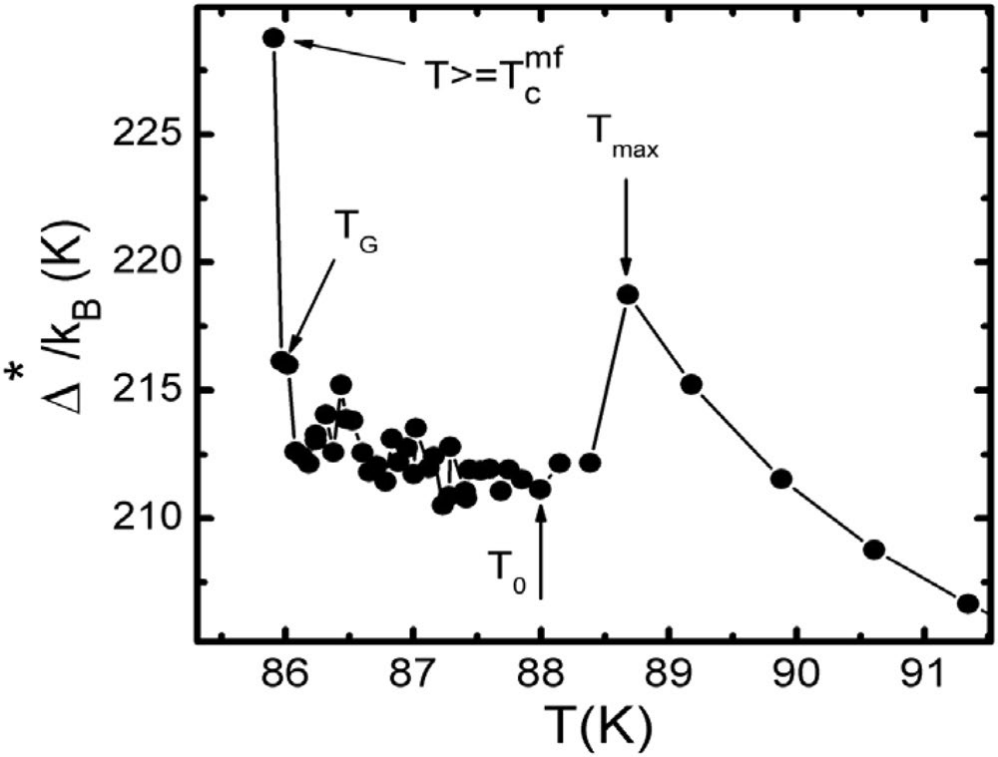
The dependence ∆*(T) in Y_0.95_Pr_0.05_Ba_2_Cu_3_O_7−δ_ single crystal near T_c_ calculated by Eq. (11) with the parameters given in the text for P = 0 (points). The arrows indicate the characteristic temperatures T_max_, T_0_, T_G_ and T_c_^mf^. The solid curve is to guide the eye.

It is seen from Fig. 7. that at P = 0 GPa the shape of the ∆*(T) curve is quite unusual, namely at T < T_pair_ = 108 K a weakly pronounced minimum is observed that corresponds to T_min_ ≈ 103 K. Below T_min_ the ∆*(T) grows uniformly showing a maximum at T_max_ = 88.7 K, where ∆*(T_max_) = 218.7 K. It is significant that this maximum is ~ 0.7 K above T_0_ = 88.0 K, which was determined from the FLC analysis (Fig. 3). With further reduction of T the ∆*(T) abruptly decreases by ~6.5 K, and ∆*(T_0_) = 212.2 K. Most likely, the observed maximum and the jump in ∆*(T) are due to the inertia of the measuring system that reflects a reaction to change in the cooling rate in the interval 93 K–88 K coinciding with the change in measurement step from δТ = 1 K at T > 93 K to δТ = 0.1 K at T < 88 K (Fig. 1). Below T_0_ there is a large scatter of experimental points, which ends with a maximum of ∆*(T_G_) = 216.1 K at T = T_G_, as shown in detail at Fig. 8. The same type voltage jumps near T_c_ were observed when studying the Hall effect on the Y_0.9_Pr_0.1_Ba_2_Cu_3_O_7−δ_ film (see Fig. 3 in^82^), which is a manifestation of specific behaviour of HTSCs containing PrBCO magnetic impurities directly inside the YBCO matrix^18,36,82^. It can also be seen that it is precisely below T_G_ the transition to the area of the critical fluctuations begins. However it is possible to obtain yet another point, which is 0.05 K above T_c_^mf^. The similar behaviour of ∆*(T) below T_max_ is also demonstrated by the single crystal under study at P = 1.7 GPa (circles at Fig. 7).

The dependences ∆*(T) obtained significantly differ from the analogous ∆*(T), which we observed in the region of SC fluctuations near T_c_ in all cuprates and pnictides studied previously^12,18,38,39,60^. For all of the mentioned HTSCs the ∆*(T) always shows a minimum with decrease of temperature at T ~ T_01_ and then a maximum at T ~ T_0_ followed by a minimum always at Т = Т_G_. We underline that the same type dependence ∆*(T) is also observed on superlattices and two-layer films of YBCO-PrBCO^23^ in which, as already mentioned, independently deposited PrBCO films do not distort the structure of YBCO layers^19^. Thus, the correlation between the temperatures T_01_, T_0_, T_G_ and the features (minima and maxima) in the ∆*(T) dependence observed in the HTSCs listed above is clearly absent in the Y_0.95_Pr_0.05_Ba_2_Cu_3_O_7−δ_ single crystal under study.

From Fig. 7 it is also seen that the pressure not only increases ∆*(T), but also changes the shape of the ∆*(T) curve, that we have never observed on pure OD YBCO single crystals^12,39^. With increasing the pressure, the maximum at T_pair_ becomes wider and is shifted to higher temperatures. Finally, at Р = 1.7 GPa the dependence ∆*(T) takes the form close to that observed for OD YBCO single crystals, demonstrating a falling linear dependence ∆*(T) in the interval Т_pair_ > T > T_01_^39^. The latter indicates a strong influence of pressure on the dynamics of the lattice^28,31,37,74^, especially in the high temperature range. Recall that T_pair_ is the temperature at which local pairs are transformed from strongly bound bosons obeying the Bose-Einstein condensation theory (BEC) into the FCPs, which obey the Bardeen-Cooper-Schrieffer theory (BCS) (refer to^5,6,8,61,83^ and references therein). In other words, this is the BEC-BCS crossover temperature predicted by the theory^84,85^ for systems with the low density of charge carriers which are cuprate HTSCs with doping less than optimal^13,60,83^. As the temperature decreases below T_01_ the ∆*(T) increases showing a maximum at T = T_max_ ≈ 93.4 K, where ∆*(T_max_) = 298.2 K (circles at Fig. 7). This is where the similarity with the Δ*(T) curve obtained for OD YBCO ends, since the non-standard behaviour of ∆*(T) begins below T_max_, which resembles the Pr effect on ∆*(T) at P = 0. In the interval T_max_ − T_0_, there is also a jump of ∆*(T), the nature of which is most likely the same as in the case of P = 0. Then, similarly to P = 0, at P = 1.7 GPa there is a region characterized by large scatter of ∆*(T) values ending with a maximum of ∆*(T_G_) = 290.0 K at T_G_ = 89.4 K.

**Figure 9:**
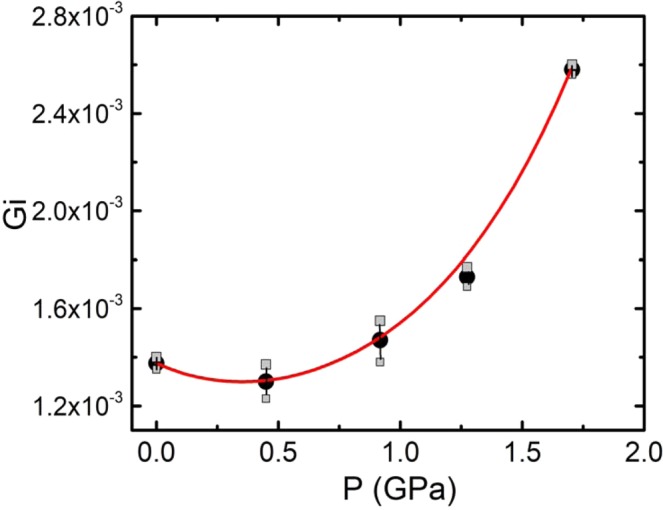
Ginzburg number Gi = (T_G_ − T_c_^mf^)/T_c_^mf^ as a function of pressure for the Y_0.95_Pr_0.05_Ba_2_Cu_3_O_7−δ_ single crystal. The red curve is an extrapolation of the experimental data by a polynomial.

Notably, in this case it is possible to confidently measure the values of Δ*(T) for three more temperatures below T_G_ (Fig. 7, circles). Moreover, the last point was obtained at the temperature of 89.18 K which is only 0.01 K above the T_c_^mf^. This is most likely to occur because the pressure, broadening the SC transition, also increases the area of critical fluctuations^24,49^. Accordingly, the Ginzburg number, Gi = (T_G_ − T_c_^mf^)/T_c_^mf^, also increases^27,46^ (refer to Fig. 9). Thus, the difference T_G_ − T_c_^mf^ in fact increases (Table 1) indicating that the genuine critical fluctuations increase with pressure^27,46^. In accordance with the anisotropic Ginzburg – Landau theory, the Ginzburg number is determined by^66,86^:

12 $$Gi=\alpha {(\frac{{k}_{B}}{\Delta c{\xi }_{c}(0){\xi }_{ab}^{2}(0)})}^{2}$$

where α is a constant of the order of 10^−3^ and Δ*c* is the jump in the heat capacity at T_c_. In accordance with the microscopic theory^86^, Δ*c* ~ T_c_N(0), where N(0) is the density of single-particle states at the Fermi level. It is assumed that Δ*c* weakly depends on P in the pressure range under consideration, since N(0), as follows from measurements of the Pauli susceptibility above T_c_, reacts weakly to a change in P (see^27,66^ and references therein).

At any rate we are interested in the value of the ratio Gi* = Gi(P)/Gi(0)^46^, which depends only on the ratio of the coherence lengths. Using the data from Tables 1 and 2 we get: Gi* ≈ 1.86 and ξ_c_(Р)/ξ_c_(P = 0) ≈ 1.17. Thus, ξ_c_(0) increases with pressure by about 17% as dξ_c_(0)/dP ≈ 0.19. Interestingly, an increase in ξ_c_(0) under pressure was also observed on YBCO^27,39^, HoBCO^46^, and HgBaCaCuO^48^. Moreover, dξ_c_(0)/dP changes in the range from 0.08^39,48^ to 0.42^46^.

At a first glance, this is an amazing result, since T_c_ simultaneously increases, and in the general theory of superconductivity it is assumed that ξ ~ 1/T_c_^63^. However an increase in ξ_c_(0) with increasing P leads to enhancing of the coupling strength between the CuO_2_ planes, J = [ξ_c_(0)/d]^2^ ^49^ namely, J(P)/J(0) ≈ 1.38, that is, the coupling strength increases by 38%. If we assume that in this case d = d_01_, which seems reasonable, then J(P)/J(0) ≈ 1.47, that is, the coupling strength between the CuO_2_ planes under pressure increases almost 1.5 times. The result obtained shows that under the influence of pressure, the simple ratio ξ_с_ ~ 1/T_c_ in cuprates is violated, and emphasizes the strong anisotropy of the conductive properties in HTSCs^13,57,60^. Considering the above results and using Eq. 12 we find ξ_ab_(0)/ξ_ab_(P) ≈ 1.26. That is to ensure the resulting increase in Gi* the ξ_ab_(0) should decrease by ≈26%.

Thus, as in OD YBCO^27,39^, in the YPrBCO single crystal under study, the pressure affects both the properties of the sample along the c-axis and the CuO_2_ conducting planes. This is in reasonable agreement with published data^27,46,66,87^, as well as with the conclusions of the general theory of superconductivity, according to which the coherence length that determines the size of the Cooper pairs (in this case it is ξ_ab_ (T)) is proportional to 1/T_c_^63^. Now, if our reasoning is correct, we can estimate the value of ξ_ab_ (P = 1.7 GPa). Taking for the HTSCs as usual ξ_ab_(0) ~ 10 ξ_c_(0) ≈ 18.4 Å (P = 0)^60^, we obtain: ξ_ab_(0) (P = 1.7 GPа) = 18.4 - (18.4 × 0.26) ≈ 13.6 Å. Such a value of ξ_ab_(0) is typical for defect-free YBCO films with doping, slightly lower than the optimum one^44,60,62^, which confirms the validity of our estimates. It remains to add that, just like the other parameters measured, the Gi(P) in YPrBCO demonstrates non-monotonic dependence on pressure. From Fig. 9. it can be seen that the character of the dependence Gi(P) changes dramatically, and again at P > 0.9 GPa. It should be noted that a similar dependence Gi(P) was observed previously^27^. The YBCO single crystals studied by Ferreira *et al*.^27^ contained a large number of twin defects. Accordingly, the pressure could minimize the influence of defects, leading to a similar dependence Gi(P). However, this question was not considered by Ferreira *et al*.^27^.

There are a number of differences we identified in the behaviour of YPrBaCuO in comparison with pure YBCO single crystals. First of all the unexpected increase in T* under pressure takes place (Fig. 7 and Table 2). In accordance with the phase diagram of cuprates^7,9,12,41^, with increasing T_c_ (in this case by application the pressure), T* should decrease, as is observed in defect-free OD and SD YBCO^38,39^ and also in HoBCO^46^ single crystals. In YPrBaCuO, an extremely low T* ~ 110 K at P = 0 (Fig. 1 and Table 2), characteristic of compounds containing Pr impurities^18^, is initially observed. This result can be explained in the assumption that the defects produced by PrBCO and PrBCO’s intrinsic magnetism effectively disturb the exchange interaction between electrons, preventing the formation of the FCPs^18,36,60^ as noted above.

Accordingly, the observed effect of increasing T* becomes clear, if we assume that pressure, improving the structure^28,31,37,74^, minimizes the effect of defects. This is confirmed by a decrease in sample resistance (Fig. 1), an increase in ΔT_fl_ and coefficient C_3D_ (Table 1), as well as an appropriate transformation of the dependence Δ*(T) (Fig. 7). Thus, the present results indicate that, under a pressure of 1.7 GPa, the YPrBaCuO under study is likely to transform into a practically defect-free YBCO single crystal, and the PG temperature is restored to T* ~ 123 K (Fig. 2 and Table 2). However, at the same time T_c_ increases by about 3 K (Table 1) which should lead to a decrease in T*, as we noted above. Thus, in this case, two opposite effects are most likely to occur: (a) an increase in T* under pressure due to minimization of the effect of additional defects, (b) a decrease in T* with an increase in T_c_ of the sample.

We can assume that if there were no effect of Pr, the YBCO single crystal under study, at P = 0 would have T* ~ 140 K, which is a typical value for OD of YBCO^72,73,77,78^. In pure OD YBCO single crystals under Р ≈ 0.95 GPa, an increase in T_c_ by only 0.7 K leads to a decrease in T* by 5 K^39^. In our case, at Р ≈ 0.95 GPa, T_c_ increases by ~2.4 K (Table 1), that is, ~3.4 times more than in an OD single crystal not containing Pr. Accordingly, the reduction of T* should be: ΔT* ≈ 3.6 × 5 = 17 K. In other words, the pressure P = 1.7 GPa, reducing the influence of defects, restores T* to the observed value T* = 140 K–17 K = 123 K, confirming the assumption made. Here we also considered that both T_c_ and T* reach saturation at P > 1 GPa (Figs. 1 and 2).

**Figure 10:**
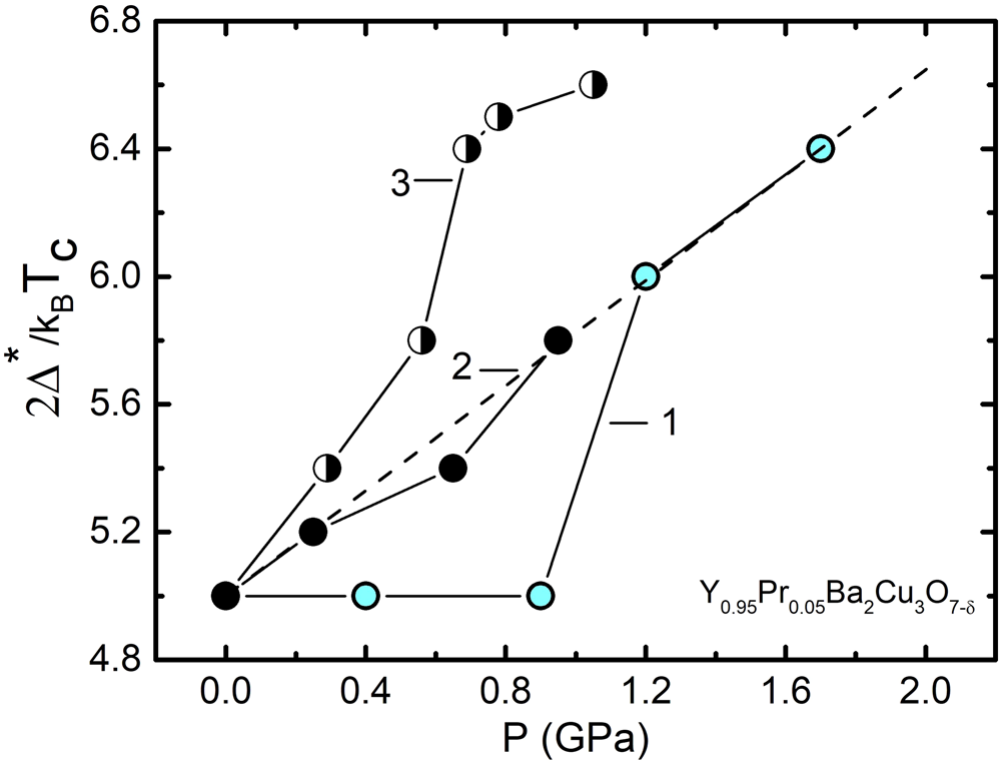
Dependences D* = 2∆*(T_G_)/k_B_T_c_ on pressure for Y_0.95_Pr_0.05_Ba_2_Cu_3_O_7−δ_ single crystals (curve 1, circles), OD YBa_2_Cu_3_O_7−δ_ (curve 2, points) and SD YBa_2_Cu_3_O_7−δ_ (curve 3, half-empty circles). All the lines are to guide the eye.

The dependence of the relation D* = 2∆*(T_G_)/k_B_T_c_ on pressure was also unusual (Fig. 10, curve 1). In contrast to OD (curve 2) and SD (curve 3) of pure YBCO single crystals, up to P ~ 0.9 GPa the D* in YPrBCO does not change, conserving the values equal to ~5 (Fig. 10 and Table 2). At Р > 0.9 GPa, a sharp increase in D* is observed, and, curiously, the dependence comes to a straight line, which is the continuation of the linear dependence D*(P) (dashed line in the figure) demonstrated by the OD single crystal. Thus, as noted above, it can be assumed that the pressure improves the structural order in the sample^28,37,40^ and minimizes the effect of defects in YPrBaCuO, the number of which at the given Pr content is probably relatively small. As a result, at Р > 1 GPa, the D*(P) dependence becomes the same as in a defect-free YBCO single crystal, where, unfortunately, the measurements were performed only up to Р = 0.95 GPa^39^. Summarising the results, we can conclude that, at P > 1 GPa, the YPrBCO single crystal behaves to a large extent as an YBCO single crystal with a relatively small number of defects. As noted above, this is confirmed by the dependence ∆*(T), measured at P = 1.7 GPa, whose shape at high T is the same as in OD YBCO single crystals (Fig. 7, circles), and also by a noticeable increase in ΔT_fl_ and C_3D_ with pressure (refer to Table 1). Attention is drawn to the fact that the growth of D* begins only after ~0.9 GPa.

In the same way increases ∆*, which measured at T_G_ (Table 2). It is curious that the same type threshold effect is observed when the slope of the σ^′−2^(T) dependences is changed (Fig. 3), so is the Ginzburg number Gi under pressure (Fig. 9), and, in fact, all of the measured parameters exhibit some threshold dependence. We believe that all the features determined are due to the specific influence of additional defects, as well as the PrBCO intrinsic magnetism. Naturally, these nontrivial results require further investigation and we expect the present study to motivate the community.

## Conclusion

For the first time, the effect of hydrostatic pressure up to 1.7 GPa on the temperature dependence of the excess conductivity σ′(T) and the PG of the Y_1-x_Pr_x_Ba_2_Cu_3_O_7-δ_ single crystal (x ≈ 0.05) was experimentally studied. It is shown that when the hydrostatic pressure is applied, the resistivity ρ(T) decreases with the rate dlnρ(100K)/dP = - (10.5 ± 0.2)% GPa^-1^, while the critical temperature increases with the rate dT_c_/dP = +1.82 K ∙ GPa^-1^ that is consistent with the literature data. It was determined that, regardless of the external pressure, the excess conductivity σ′(T) in the interval T_c_ < T < T_01_ is described by the classical fluctuation theories, namely the 3D Aslamazov-Larkin theory (Eq. 4) and Lawrence-Doniach (LD) theory (Eq. 6). The fluctuation contribution of the Maki-Thompson (Eq. 7) is not observed, which indicates the presence of defects in the sample induced by PrBCO cells embedded in the YBCO matrix. Moreover, the dopant has intrinsic magnetic moment μPrBCO ≈2μB.

The presence of defects led to the observation of a number of unusual effects. Among them is a small value of the region of SC fluctuations, ΔT_fl_ = 6.8 K, and a very small (for the cuprates with T_c_ = 85.2 K) value of T* = 110 K. It is assumed that the defects produced by PrBCO as well as its intrinsic magnetism effectively disturb the exchange interaction between electrons, preventing the formation of the FCPs. Under pressure, T* increases to ~ 123 K. Such behaviour of T* is unusual as according to the phase diagram of cuprates, the T* should decrease with increasing T_c_. The observed effect of increasing T* becomes clear, if we assume that pressure, improving the structure, minimizes the effect of defects and restores the value of T*. This is confirmed by a decrease in sample resistance (Fig. 1), a noticeable increase in ΔT_fl_ and a C_3D_ coefficient (Table 1), as well as a corresponding transformation of the dependence Δ*(T) (Fig. 7). Simultaneously, the pressure reduces the distance between the conducting layers d_01_, but increases ξ_c_(0) (Table 1). A decrease in d_01_ seems reasonable, since the pressure reduces all the dimensions of the unit cell. The increase in ξ_c_(0) is also observed in a number of other works, and is explained when analyzing the Ginzburg parameter [(Eq. (12)].

It is shown that the shape of the dependence of the PG parameter, Δ*(T), calculated according to Eq. (11), at Р = 0 is rather unusual, with a weakly pronounced minimum at 103 K = Tmin <Tpair = 108 K (Fig. 7). The pressure noticeably changes the shape of the Δ*(T) curve, which is not observed on pure OD YBCO single crystals. At Р = 1.7 GPa, the shape of Δ*(T) becomes the same as in OD of YBCO, confirming the assumption about minimization of the effect of defects under pressure. At the same time, there is an increase in Δ*(T) and D* (Fig. 7) as dlnΔ*/dP = 0.17 GPa^-1^, which, however, is almost two times less than dlnΔ*/dP= 0.32 GPa^-1^ measured for OD YBCO single crystal. As is known, the change in the parameters of cuprates under the action of hydrostatic pressure, and above all, the growth of Tc can occur both due to an increase in the density of charge carriers nf in the CuO_2_ planes, and due to the convergence of peaks of the density of states. Considering the present results, it can be concluded that in YPrBCO, the influence of both these processes is relatively small due to the likely localization of part of the charge carriers, the presence of defects and the intrinsic magnetism of PrBCO.

We note more nontrivial results that are not observed on the pure YBCO single crystals. These are the nonlinear character of the dependences of Tc and ΔTfl on P and a sharp change in the character of the pressure dependences ρ(100 K) and T* at T ≥ 0.9 GPa (Fig. 2). Moreover, the change of virtually all measured parameters with pressure has a threshold character. Thus, the slope of the dependences σ′-2(T) (Fig. 3), the growth of σ′(T), the shape of the curves ln(σ′) vs (1/T) (Fig. 6) and the Ginzburg parameter (Fig. 9) begin change only at Р ≥ 0.9 GPa. This effect is most clearly observed in the D*(P) dependence (Fig. 10), which is determined with the highest accuracy. Unlike the OD and SD pure YBCO single crystals (curves 2 and 3 in Fig. 10), D* in YPrBCO does not change up to P ~ 0.9 GPa, keeping the value equal to ~5 (Table 2). At Р > 0.9 GPa, a sharp increase in D* is observed, and the points fall on a straight line, which is a continuation of the D*(P) dependence in the defect-free OD YBCO single crystal. This result confirms the assumption that pressure improves the structural order in the sample and, thus, minimizes the effect of defects in Y0.95Pr0.05Ba2Cu3O7-δ. To conclude all the features found are due to the specific influence of additional defects produced by Pr, as well as by the intrinsic magnetism of PrBCO. These non-trivial results require further study.

## Experimental Methodology

Y_1−х_Pr_x_Ba_2_Cu_3_O_7−δ_ single crystals were grown by the solution-melt technology^88^, as described in previous studies^34,35,89^. Y_2_O_3_, BaCO_3_, CuO were used as initial components for growing the crystals. However, the use of BaCO_3_ requires preliminary high-temperature annealing of the stock for decarbonization of barium carbonate. To dope the Y sites with Pr, the Pr_5_O_11_ was added to the initial stock in an appropriate percentage. The regimes of growing and saturating crystals with oxygen were the same as described elsewhere^34,35,90^.

Rectangular crystals of about 3 × 0.5 × 0.3 mm^3^ (0.3 mm corresponds to the c-axis) were chosen from the same batch for the resistivity measurements. Samples with unidirectional twin boundaries were obtained and a bridge ~0.2 mm wide and with 0.3 mm spacing between pairs of electrical contacts was cut from the crystal. In this case, the experimental geometry was chosen in such a way that the vector of the transport current I along the bridge was parallel to the twinning planes (I || TB)^12,35,91^. An automated setup engulfing the four-point probe technique (here stabilized measuring current of up to 10 mA) was employed to determine the ab-plane resistivity, ρ_ab_(T)^46,92^. The silver epoxy contacts were glued to the end points of the crystal aiming to form a uniform current distribution in the central region where voltage probes in the form of parallel stripes were placed. Contact resistances below 1 Ω were obtained.

The sample morphology, the arrangement of contacts and other details are discussed in previous work^29,46^. Temperature measurements were performed with a platinum sensor with an accuracy of about 1 mK. The hydrostatic P was generated inside a Teflon cup in a copper–beryllium piston-cylinder cell, as described in previous work^93^. The applied pressure was measured using a manganin gauge made of a 25 Ω wire. Transformer oil acted as the transmitting medium and pressures were changed at room temperature in the order of increasing magnitude. At every P, experimental measurements were performed at rates of about 0.1 K/min near T_c_ and 0.5 K/min at T ≫ T_c_.
